# Quercetin Stimulates Bone Marrow Mesenchymal Stem Cell Differentiation through an Estrogen Receptor-Mediated Pathway

**DOI:** 10.1155/2018/4178021

**Published:** 2018-03-15

**Authors:** Xin-Gang Pang, Yu Cong, Ni-Rong Bao, Yong-Gang Li, Jian-Ning Zhao

**Affiliations:** ^1^Department of Orthopedics, Jinling Hospital, School of Medicine, Nanjing University, Zhongshan East Road, No. 305, Nanjing 210002, China; ^2^Southeast University School of Medicine, Dingjiaqiao Road, No. 87, Nanjing 210009, China; ^3^Department of Orthopedics, Zhongda Hospital, School of Medicine, Southeast University, Dingjiaqiao Road, No. 87, Nanjing 210009, China

## Abstract

**Objectives:**

The present study aimed to investigate the overall effect of quercetin on mouse bone marrow mesenchymal stem cell (BMSC) proliferation and osteogenic differentiation* in vitro*.

**Materials and Methods:**

BMSCs were treated with different concentrations of quercetin for 6 days. The effects of quercetin on cell proliferation were assessed at predetermined times using Cell Counting Kit-8 (CCK-8) assay. The cells were then treated with quercetin, estrogen, or an estrogen receptor (ER) antagonist (which was also administered in the presence of quercetin or estrogen) for 7 or 21 days. The effects of quercetin on BMSC osteogenic differentiation were analyzed by an alkaline phosphatase (ALP) assay kit, Alizarin Red S staining (ARS), quantitative real-time PCR (qPCR), and western blotting.

**Results:**

The CCK-8 and ALP assays and ARS staining showed that quercetin significantly enhanced BMSC proliferation, ALP activity, and extracellular matrix production and mineralization, respectively. The qPCR results indicated that quercetin promoted osterix* (OSX)*, runt-related transcription factor 2* (RUNX2)*, and osteopontin* (OPN)* transcription in the presence of osteoinduction medium, and the western blotting results indicated that quercetin enhanced bone morphogenetic protein 2 (BMP2), Smad1, Smad4, RUNX2, OSX, and OPN expression and Smad1 phosphorylation. Treatment with the ER inhibitor ICI182780 blocked the effects of quercetin.

**Conclusions:**

Our data demonstrated that quercetin promotes BMSC proliferation and osteogenic differentiation. Quercetin enhances BMP signaling pathway activation and upregulates the expression of downstream genes, such as* OSX*,* RUNX2*, and* OPN*, via the ER.

## 1. Introduction

Mesenchymal stem cells are a class of cells that exist in multiple organs and retain the capacity for self-renewal and differentiation into multilineage tissues for life [1, 2]. Bone marrow mesenchymal stem cells (BMSCs), which are isolated from the bone marrow, are regarded as seed cells for bone tissue engineering because of their great multidirectional differentiation ability and reproductive activity [3–5]. BMSCs reportedly give rise to osteogenic, chondrogenic, and adipogenic cells, which are widely present in the bone marrow and cancellous bone and play an important role in bone metabolism. Due to these properties, BMSCs are a potential source of cells for cell and gene therapy experiments and are widely used in studies of bone regeneration [6, 7].

Estrogen is the key regulator of bone metabolism [8]. Menopause and the accompanying loss of ovarian estrogen are associated with declines in bone mineral density [9]. The protective effects of estrogen on bone in postmenopausal women were initially thought to be mediated by the suppression of bone resorption. However, recent studies have demonstrated that in addition to inhibiting osteoclast development, estrogen also promotes osteogenesis [10, 11]. Khosla et al. proposed that estrogen decreases bone resorption and maintains bone formation by exerting direct effects on osteocytes, osteoblasts, and osteoclasts [12], a process that requires the participation of the estrogen receptors (ERs) expressed on osteoblasts [13–15]. Bone morphogenetic protein (BMP), a pleiotropic cytokine belonging to the TGF-*β* super family, has osteogenic properties. Runt-related transcription factor 2 (RUNX2) and osterix (OSX) are the transcription factors required for osteoblastogenesis. BMP binds heterodimeric receptors to activate Smad proteins, which transactivate osteoblastogenic genes either directly or via RUNX2/OSX [16, 17]. These pathways do not work independently. Researchers have noted that strong cross-talk occurs among BMP, estrogen, and other signaling pathways. Previous studies have indicated that estrogen upregulates BMP2 expression and enhances BMP signaling pathway activation [10, 18].

Quercetin is one of the principal flavonoids and the most widely studied product of its type because of its pharmacological properties and beneficial health effects [19]. A variety of studies have begun amassing evidence regarding the anti-inflammatory [20], anticarcinogenic [21], neuroprotective, cardioprotective, and chemopreventive effects of quercetin [22]. Horcajada-Molteni et al. [23] demonstrated that rutin, a glycoside derivative of quercetin, inhibits ovariectomy-induced osteopenia in female rats. Moreover, several recent studies [6, 19, 24, 25] have indicated that quercetin promotes osteoblast differentiation. However, the mechanism by which quercetin induces osteogenic differentiation remains unclear.

Flavonoids, which are widely used in traditional Chinese medicine [17], chemically resemble estrogen. Some flavonoids have been used as estrogen substitutes, and several flavonoids, including icariin [26], genistein [27], and kaempferol [28], have been shown to promote osteogenic differentiation via their estrogen effects. As the structures of these flavonoids are similar to that of quercetin [29], we surmised that quercetin also has estrogenic effects on BMSCs and promotes osteogenic differentiation via the ER. In the present study, we investigated the estrogenic and osteogenic effects of quercetin on cultured BMSCs and elucidated the mechanisms by which the flavonoid exerts its effects. We compared ALP activity levels, OSX and OPN transcription levels, and osteogenic differentiation protein marker expression levels among quercetin-, estrogen-, and ER antagonist (ICI182780)-treated groups after osteogenic induction to analyze the effects of quercetin on cell proliferation.

## 2. Materials and Methods

### 2.1. BMSC and Quercetin Preparation

A frozen vial of mouse BMSCs isolated from C57BL/6 mice was purchased from Cyagen Biosciences Inc. (Guangzhou, China). The cells were thawed and then expanded in the complete medium (CM), that is, DMEM (Sigma–Aldrich, St. Louis, MO, USA) supplemented with 10% fetal bovine serum (FBS) (Elite Biotech, Heidelberg, GER) and 1% penicillin–streptomycin solution (Sigma–Aldrich, St. Louis, MO, USA), at 37°C in a humidified atmosphere of 95% air/5% CO2.

Quercetin was purchased from the National Institute for the Control of Pharmaceutical and Biological Products (Beijing, China) and was dissolved in DMSO (Sigma–Aldrich, St. Louis, MO, USA) to obtain a 20 mM stock solution, which was ultimately diluted in medium to obtain solutions of the desired concentrations. The control group was treated with additional DMSO to eliminate the effect of the reagent on the results. The final concentration of DMSO in the control and experimental groups was 0.1%.

### 2.2. Cell Proliferation Assay

Cell Counting Kit-8 (CCK-8) (Nanjing Jiancheng Bioengineering Institute, Nanjing, China) was used to determine the effect of quercetin on cell proliferation. BMSCs were seeded in 96 well plates at a density of 2*∗*10^∧^4 cells/well and cultured in CM. The cells were cultured for 24 hours, after which they were treated with quercetin at the indicated concentrations (0-, 0.1-, 1-, 2.5-, and 5-*μ*M) before being cultured for an additional 6 days. The 0* μ*M group, which was treated with DMSO, served as a control group. The cells were treated with 100* μ*l of fresh medium and 10* μ*l of CCK-8 solution at predetermined time and then incubated for 2 h. Enzyme-linked immunosorbent assay (ELISA) was used to measure the optical density (OD) values at a wavelength of 450 nm. The relative cell proliferation rate was calculated using the mean rate of the control group.

### 2.3. Alkaline Phosphatase (ALP) Activity Assay

ALP activity was evaluated using a 4-nitrophenyl phosphate colorimetric assay. BMSCs were seeded in 6-well plates at a density of 1*∗*10^∧^5 cells/well and cultured in CM until they reached 60% confluence. The cells were then divided into the following seven experimental groups: a negative control group (control), five quercetin-treated groups, and one estrogen-treated group. Cells cultured in CM alone served as a negative control group, and cells cultured in OIM with 10 nM 17ß-estrogen (E2) (Sigma–Aldrich, St. Louis, MO, USA) served as a positive control group. The BMSCs in the quercetin-treated groups were cultured in OIM containing 0-, 0.1-, 1-, 2.5-, and 5-*μ*M quercetin, respectively, for 7 days. We made OIM by dissolving dexamethasone (10 nM), ascorbic acid (50* μ*g/ml), and ß-glycerophosphate (10 mM) (Sigma–Aldrich, St. Louis, MO, USA) in CM. ALP activity was quantified in cell lysates using an ALP assay kit (Nanjing Jiancheng Bioengineering Institute, Nanjing, China), according to the manufacturer's instructions. Each experiment was repeated twice; thus, the results are the means of three experiments.

### 2.4. Matrix Mineralization Assay

Extracellular matrix mineralization was observed by Alizarin Red S (ARS) staining. As in the ALP assay, BMSCs were seeded in 6-well plates at a density of 1*∗*10^∧^5 cells/well. After reaching confluence, the cells were incubated in CM or OIM containing quercetin or estrogen for 21 days. At the end of the incubation period, the cells were washed with phosphate-buffered saline (PBS) and fixed in 4% polysorbate for 4 h. The cells were then incubated with ARS (0.4%, pH 4.2, Sigma) for 30 min at room temperature.

### 2.5. Quantitative Real-Time PCR (qPCR)

The effects of quercetin and E2 on* RUNX2*,* OSX, *and* OPN* mRNA expression were investigated by real-time PCR. The cells were treated with the above reagents in the absence or presence of ICI182780 (an ER inhibitor) for 7 days, after which total RNA was extracted with TRIzol reagent (Invitrogen Corp., Carlsbad, CA, USA), and cDNA was prepared with a Transcriptor First-Strand cDNA Synthesis Kit (Thermo Fisher Scientific Inc., Waltham, MA, USA). For real-time PCR, a 20* μ*l reaction mixture containing 10* μ*l of SYBR Green Real-time PCR Master Mix (Toyobo Biotech Co., Ltd., Osaka, JP), cDNA, and primers was thermocycled in an ABI Step One Plus Real-time PCR System Thermal Cycler (Applied Biosystems Inc., Carlsbad, CA, USA). The primers, whose sequences are shown in Table 1, were purchased commercially (GeneScript Co., Ltd., Nanjing, China). The expression of* RUNX2*,* OSX, *and* OPN* gene was evaluated according to the threshold cycle (Ct) values and was normalized to glyceraldehyde-3-phosphate dehydrogenase (GAPDH) gene expression.

### 2.6. Western Blot Analysis

The ER-mediated effects of quercetin on BMP signaling pathway activation and maker protein expression were investigated by western blotting analysis. BMSCs were treated with 2.5* μ*M quercetin or vehicle control in the absence or presence of 1* μ*M ICI182780 for 7 days, after which the expression of BMP2, Smad1, p-Smad1, Smad4, RUNX2, OSX, and OPN protein was assessed. For western blotting analysis, the cells were lysed in cold Nonidet P-40 (NP-40) lysis buffer (pH 7.6) containing 50 mM Tris–HCl, 150 mM NaCl, 10% glycerol, 1% NP-40, 1 mM phenylmethylsulfonyl fluoride, 1* μ*g/ml leupeptin, 1* μ*g/ml aprotinin, and 1* μ*g/ml pepstatin for 15 min at 4°C. The cell lysates were collected by scraping and then centrifuged at 14,000*g* for 10 min. Protein concentrations were assessed using BCA protein assay reagent (Jiangsu Keygen Biotech Corp., Nanjing, China). Twenty micrograms of whole-cell lysates was electrophoresed on an SDS-polyacrylamide gel before being transferred to a PVDF membrane (Jiangsu Keygen Biotech Corp., Nanjing, China), which was blocked in PBS containing 6% low-fat milk and 0.1% Tween 20 (PBST). The blots were then incubated with RUNX2-, BMP2-, Smad1-, p-Smad1-, Smad4-, OSX-, or OPN-antibodies (Jiangsu Keygen Biotech Corp., Nanjing, China), washed twice with PBST, and probed with horseradish peroxidase-conjugated goat anti-mouse secondary antibodies. The protein bands were visualized using a G:BOXChemiXR5 System (Syngene, Cambridge, UK), and the western blotting results were quantified using Gel-Pro32 (Media Cybernetics).

### 2.7. Statistical Analysis

Quantitative data are expressed as the mean ± standard deviation (SD) of three separate experiments and were analyzed by one-way ANOVA using SPSS 18.0. *P* values less than 0.05 were considered significant.

## 3. Results

### 3.1. Quercetin Enhanced Cell Proliferation

**Figure 1:**
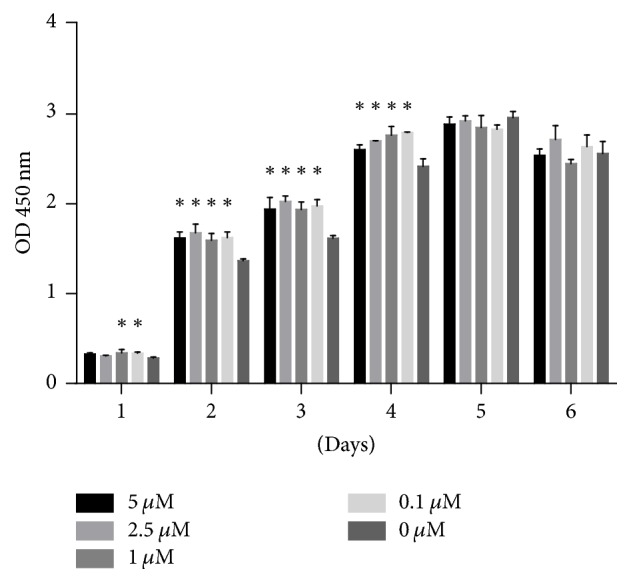
Effect of quercetin on cell proliferation in BMSCs. Cell proliferation was expressed using OD, which was measured for 6 consecutive days. Each value represents the mean ± SD from triplicate experiments. *∗* indicates a significant difference between the control and test groups at *P* < 0.05.

Cell proliferation was assessed using CCK-8 assay, as shown in Figure 1. The ODs in the control and quercetin groups increased during the first 5 days of the experiment and then decreased on day 6 of the experiment. The ODs in the 0.1* μ*M and 1* μ*M quercetin groups increased significantly compared with those in the control group on the first day of the experiment. We noted no significant differences in OD between the 2.5* μ*M and 5* μ*M quercetin groups and the control group. Treatment with 0.1* μ*M–5* μ*M quercetin significantly stimulated cell proliferation compared with treatment with the control on days 2, 3, and 4 of the experiment.

### 3.2. Quercetin Enhanced Osteoblastic Differentiation and Extracellular Matrix Production and Mineralization

**Figure 2:**
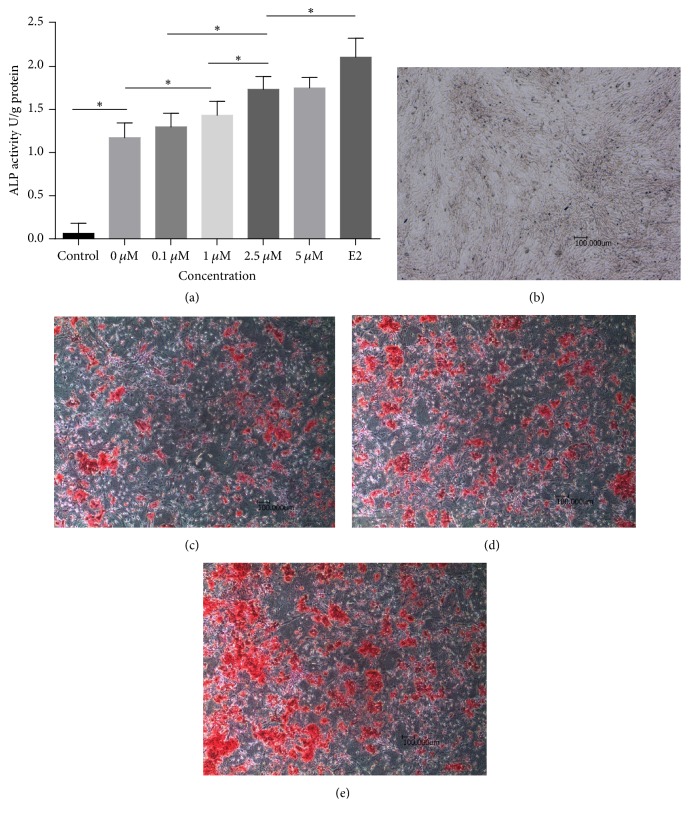
Effects of quercetin on ALP activity (a) and matrix mineralization (b)–(e). (a) For the ALP activity assay, cells were incubated in CM or OIM with or without quercetin or E2 for 7 days. Each value represents the mean ± SD from triplicate experiments. *∗* indicates a significant difference between the two groups at *P* < 0.05. (b)–(e) For the calcium nodule assay, the cells were incubated in CM (b), OIM with DMSO (c), OIM with quercetin (d), or OIM with E2 (e) for 21 days.

Quantitative analysis of ALP activity was performed using an Alkaline Phosphatase Assay Kit. ALP activity was significantly enhanced in the 0* μ*M quercetin group compared with the control group, in which the cells were cultured in CM, on day 7 of the experiment. Treatment with additional quercetin or E2 further enhanced ALP activity. As shown in Figure 2(a), quercetin (0* μ*M–5* μ*M) induced osteoblastic differentiation in a dose-dependent manner. Notably, treatment with 5* μ*M quercetin had the greatest effect on ALP activity; however, we noted no significant difference in ALP activity between the 2.5* μ*M and 5* μ*M quercetin groups. Thus, 2.5* μ*M quercetin was used for subsequent experiments. Additionally, E2 (10 nM) was successfully utilized as a positive control in this experiment, as it also increased cell ALP activity.

The effects of quercetin on extracellular matrix production and mineralization were further investigated by ARS staining for calcium deposits. As shown in Figures 2(b)–2(e), red (positive)-stained bone nodules were noted in the 0* μ*M quercetin group; however, no bone nodules were noted in the control group. Treatment with 10 nM E2 or 2.5* μ*M quercetin resulted in the formation of a large number of mineralized bone nodules. In addition to increasing the number of bone nodules, the above treatments also increased the area of each nodule. Some of the nodules joined together, forming large irregularly shaped nodules that stained a deep red.

### 3.3. Quercetin Upregulates Osteoblast-Specific Marker Gene Expression

**Figure 3:**
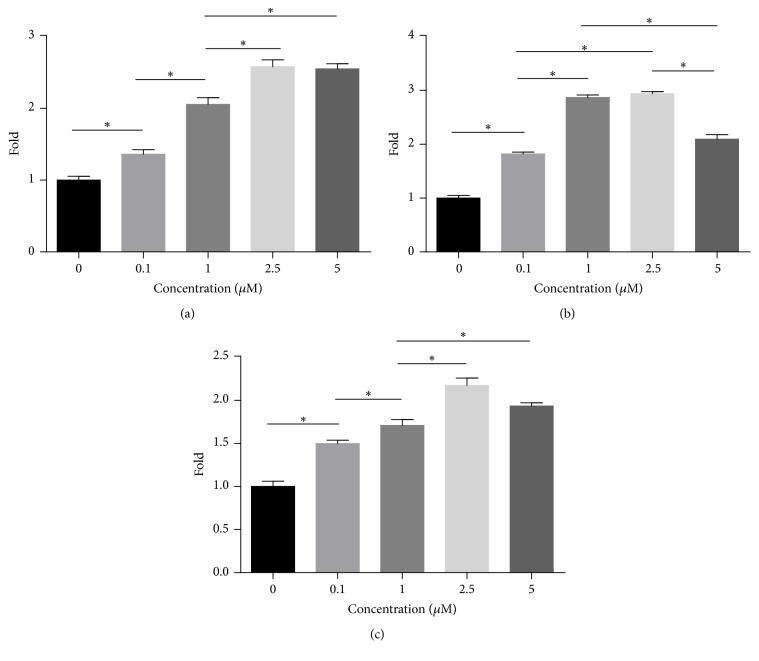
*RUNX2*,* OSX*, and* OPN* mRNA expression in BMSCs treated with quercetin. (a)–(c) The cells were incubated in osteogenic-inducing medium (OIM) with different concentrations of quercetin for 7 days. mRNA expression was then analyzed by real-time PCR. Target gene mRNA expression levels were normalized to GAPDH mRNA levels. Each value represents the mean ± SD from triplicate experiments. *∗* indicates a significant difference between the two groups at *P* < 0.05.

As shown in Figure 3, treatment with 0.1–5* μ*M quercetin upregulated* RUNX2*,* OSX*, and* OPN *expression by 1.36–2.58-, 1.82–2.94-, and 1.50–2.17-fold, respectively, compared with treatment with the DMSO (0* μ*M quercetin group). These results were consistent with those of the ALP activity assay, suggesting that quercetin stimulated BMSC differentiation and function by upregulating* RUNX2, OSX*, and* OPN* expression. Treatment with quercetin at the indicated concentration (2.5* μ*M) elicited the maximal increases in the expression of the above proteins. Thus, this concentration was used for subsequent experiments.

### 3.4. Quercetin Regulates Osteoblast-Specific Gene Expression via the ER

**Figure 4:**
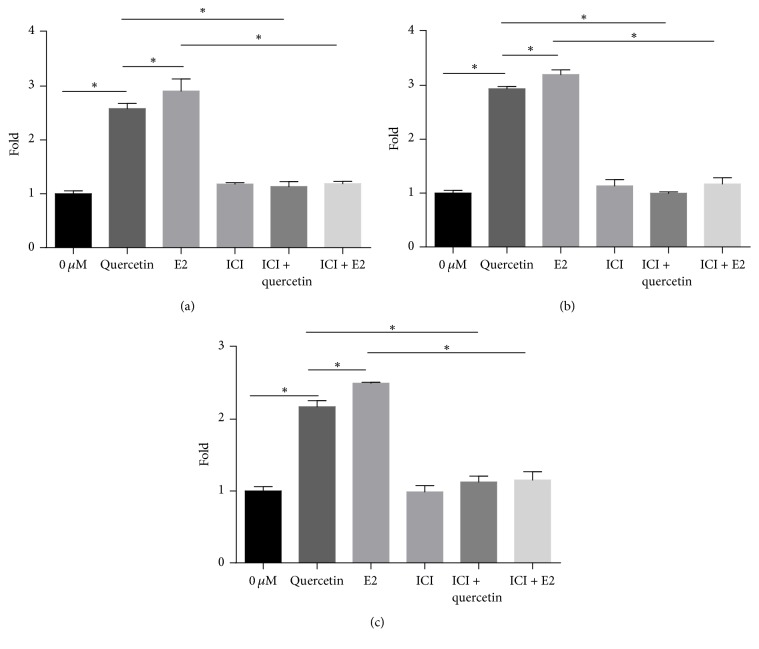
Effects of ICI182780 on* RUNX2*,* OSX*, and* OPN* mRNA expression in quercetin-treated BMSCs. The cells were cotreated with quercetin or E2 and 1 *μ*M ICI182780 for 7 days. Each value represents the mean ± SD from triplicate experiments. ^*∗*^Significant differences between the values at *P* < 0.05.

As shown in Figure 4, treatment with 2.5* μ*M quercetin and 10 nM E2 upregulated* RUNX2, OSX*, and* OPN* expression compared with treatment with DMSO (0* μ*M quercetin group). The addition of 1* μ*M ICI182780, a high-affinity ER antagonist, inhibited* RUNX2, OSX*, and* OPN *expression.* RUNX2*,* OSX*, and* OPN *expression levels were slightly different between the group treated with E2 and ICI182780 and the group treated with ICI182780 alone; however, there were no significant differences in* RUNX2*,* OSX*, and* OPN *expression between the two groups. Similarly, there were no significant differences in* RUNX2*,* OSX*, and* OPN *expression between the group treated with quercetin and ICI182780 and that treated with ICI182780 alone. The upregulatory effects of both quercetin and E2 were blocked by ICI182780.

### 3.5. Quercetin Regulates Osteoblast Marker Protein Expression

**Figure 5:**
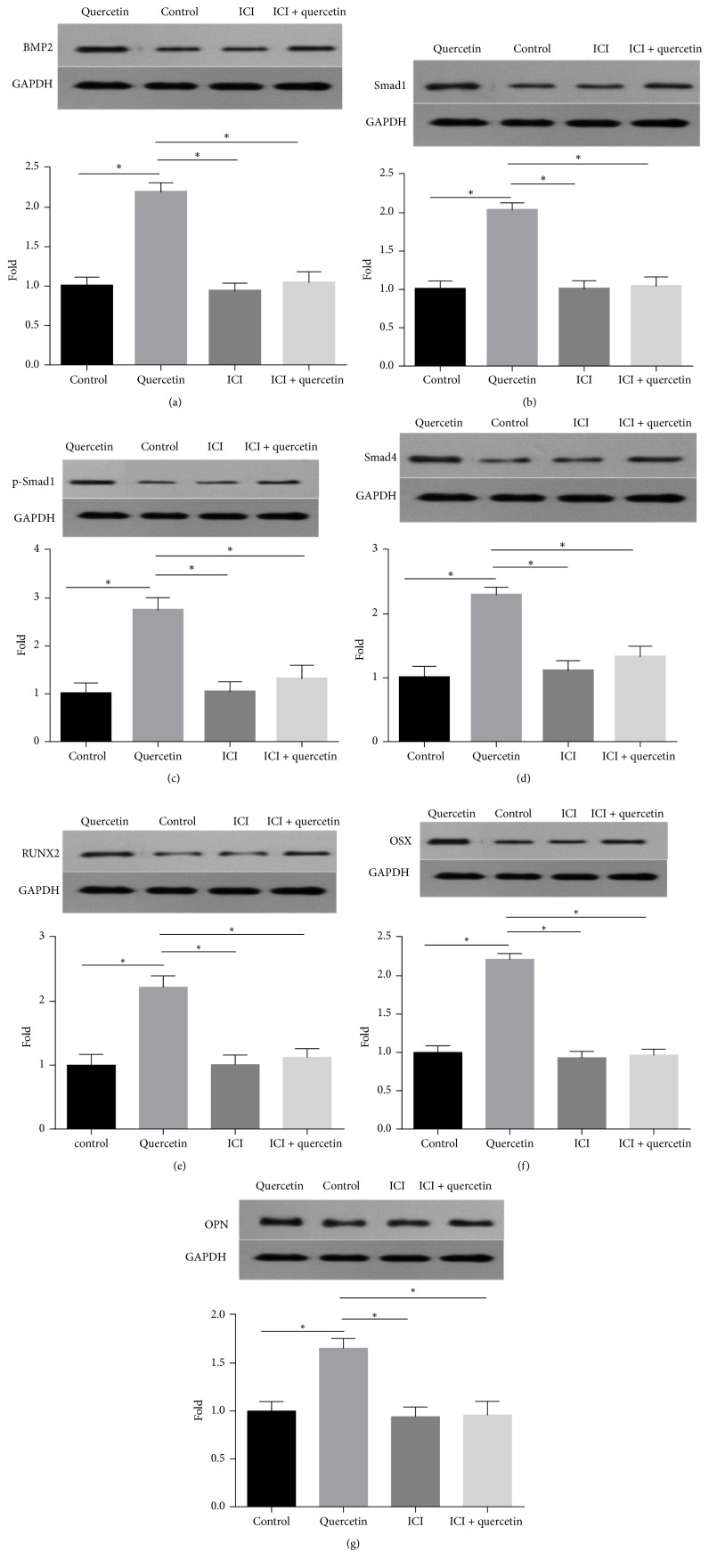
Effects of ICI182780 on BMP2, Smad1, p-Smad1, Smad4, RUNX2, OSX, and OPN expression in quercetin-treated BMSCs. The cells were cotreated with quercetin and 1 *μ*M ICI 182780 for 7 days, after which the expression levels of the above proteins were analyzed by western blotting. The data are presented as the ratio of protein expression in the treated group to protein expression in the control group. Each value represents the mean ± SD from triplicate experiments. ^*∗*^Significant differences between the values at *P* < 0.05.

As shown in Figure 5, BMP2, Smad1, p-Smad1, Smad4, RUNX2, OST, and OPN expression levels in the 2.5* μ*M quercetin group were upregulated (2.19-, 2.03-, 2.76-, 2.29-, 2.22-, 2.22-, and 1.66-fold, respectively) compared with those in the control group. The relative expression levels of these proteins in the group treated with 2.5* μ*M quercetin and 1* μ*M ICI182780 were 1.040-, 1.039-, 1.312-, 1.326-, 1.123-, 0.965-, and 0.960-fold higher than those in the control group, respectively, indicating the fact that the ER is involved in mediating the effects of quercetin.

## 4. Discussion

BMSCs are regarded as seed cells for bone tissue engineering because of their great multidirectional differentiation ability and reproductive activity. BMSCs reportedly give rise to several cells, including osteoblasts [3–5], and play a crucial role in the achievement and maintenance of an appropriate bone mass. Thus, it is important to promote BMSC proliferation and osteogenic differentiation.

In the current study, we first evaluated the effect of quercetin on BMSC proliferation. Our results showed that quercetin significantly increased BMSC proliferation in the quercetin-treated groups compared with the control group. ALP [30] is an early osteogenic marker and plays an important role in bone formation. A peak in ALP activity is indicative of differentiation [31, 32]. We quantified ALP activity to evaluate the effect of quercetin on BMSC osteogenic differentiation. The results indicated that quercetin significantly increased ALP activity on day 7 of the experiment and that 2.5* μ*M and 5* μ*M were the most effective quercetin concentrations utilized herein. We validated the above findings by ARS staining [33, 34]. The results showed that quercetin enhanced extracellular matrix formation.

RUNX2 is a core transcription factor that plays a pivotal role in regulating osteogenic differentiation. OSX, a zinc finger-containing transcription factor which is required for osteoblastic differentiation, acts as a downstream factor of RUNX2 [35–37]. OPN, an acidic glycoprotein that binds with hydroxyapatite in bone extracellular matrix [38], is a direct downstream target of RUNX2 [39]. In this study, we found that quercetin significantly increased* RUNX2*,* OSX*, and* OPN* mRNA expression levels. These results suggested that quercetin enhances BMSC osteogenic differentiation by regulating the expression of the transcription factors RUNX2 and OSX.

We then sought to determine how quercetin regulates the expression of the two transcription factors.

Estrogen is the key regulator of bone metabolism* in vivo*. Menopause and the accompanying loss of ovarian estrogen are associated with declines in bone mineral density [9]. Recent studies suggest that estrogen participates in the preservation of bone mass through non-nucleus-initiated mechanism via ER alpha [40]. A series of scientific studies showed that estrogen regulates bone metabolism by regulating several signaling molecules or interacting with other signaling pathways, such as the BMP/Smad signaling pathways [41–44]. In the current study, we confirmed that E2 enhances ALP activity and extracellular matrix formation and upregulates RUNX2 and OSX expression. In addition, quercetin, which is a member of a class of flavonoids that chemically resemble estrogen [28], has been shown to have biologic characteristics similar to those of estrogen in many studies regarding breast cancer [45–47]. Thus, we hypothesized that quercetin upregulates RUNX2, OSX, and OPN expression and promotes osteogenic differentiation by regulation of the estrogen signaling pathway. To confirm this hypothesis, we performed an experiment in which we investigated whether the stimulatory effects of quercetin on osteogenic differentiation in BMSCs were abolished by an ER inhibitor (ICI182780). The results showed that ICI182780 significantly reduced RUNX2, OSX, and OPN expression, indicating that ICI182780 blocked the upregulatory effects of quercetin and E2 on RUNX2, OSX, and OPN gene expression. These data clearly demonstrated that the estrogen signaling pathway plays a crucial role during quercetin-induced osteogenesis in BMSCs.

BMPs are members of the TGF-ß superfamily that were originally identified in bone-inductive extracts capable of inducing bone formation at ectopic sites [48, 49]. It is generally accepted that BMPs transmit signals to Smad proteins by binding to BMP receptors to ultimately regulate the transcription of RUNX2, OSX, and other genes [48]. As the most famous member of the BMP family, BMP2 has been shown to stimulate osteoblast differentiation and osteogenic nodule formation* in vitro *and bone formation* in vivo *[50–52]. The effect of BMP2 on osteogenic differentiation is enhanced by estrogen [10, 18], phytoestrogens [27], and flavonoids, such as genistein and icariin [26]. Other studies have shown that estrogenic compounds enhance bone formation by increasing BMP2 gene transcription [10, 18].

Quercetin is similar to genistein and icariin, as it is also a plant flavonoid and has estrogenic effects. Thus, we hypothesized that quercetin enhances BMP signaling pathway activation via the ER. First, to determine whether the BMP signaling pathway is also involved in quercetin-mediated BMSC osteogenic differentiation, we incubated cells with quercetin and OIM, after which we assessed protein marker expression using western blotting. Second, to confirm our hypothesis, we investigated whether the stimulatory effects of quercetin on osteogenic differentiation in BMSCs were abolished by the inhibitor ICI182780. Consistent with the results of previous studies, our results showed that quercetin significantly upregulated BMP2, Smad1, p-Smad1, Smad4, RUNX2, OSX, and OPN expression and that this upregulatory effect was blocked by ICI182780. Taken together, these findings showed that the E2 and BMP/Smad signaling pathways played important roles in the effects of quercetin on BMSC differentiation into osteoblasts.

Quercetin, a flavonoid, chemically resembles estrogen [28, 45–47]. Previous studies indicated that strong cross-talk occurs between the BMP and estrogen signaling pathways [18, 53]. In the present study, we found that quercetin stimulated BMP signaling by interacting with the ER, which led to increases in the expression of the downstream transcription factors RUNX2 and OSX and ultimately stimulated BMSC differentiation and mineralization.

## Figures and Tables

**Table 1:** Sequences of the primers of the target and housekeeping genes used for RT-PCR.

	Sequence (5′-3′)	Length of product
GAPDH	Sense primer: 5-GGCCTTCCGTGTTCCTACC-3	103 bp
	Antisense primer: 5-TGCCTGCTTCACCACCTTC-3	
RUNX2	Sense primer: 5-CCAAGTAGCCAGGTTCAACG-3	64 bp
	Antisense primer: 5-GGTGAAACTCTTGCCTCGTC-3	
OSX	Sense primer: 5-CTTTCGTCTGCAACTGGCTT-3	123 bp
	Antisense primer: 5-TAAAGCGCTTGGAACAGAGC-3	
OPN	Sense primer: 5-CAGCCATGAGTCAAGTCAGC-3	116 bp
	Antisense primer: 5-TGTGGCTGTGAAACTTGTGG-3	
